# A Hospitalized Patient With Systemic Scleroderma Requiring ICU-Level Care

**DOI:** 10.7759/cureus.111920

**Published:** 2026-07-01

**Authors:** Darius Ghahary, Bryan Goldstein, Linda Hong, Matthew Maffey

**Affiliations:** 1 Family Medicine, Morristown Medical Center, Morristown, New Jersey, USA

**Keywords:** dysphagia assessment, flash pulmonary edema, interstitial lung diseases (ilds), outcomes of hypertensive emergency, pneumonia hospitalization, scleroderma renal crises, systemic scleroderma, systemic sclerosis interstitial lung disease (ssc-ild)

## Abstract

A patient with a history of systemic scleroderma complicated by interstitial lung disease on two liters of home oxygen at baseline presented to the emergency department with a two-week history of progressively worsening dysphagia. An initial chest X-ray showed concerns for bilateral pneumonia and aspiration. Initial laboratory results revealed anemia with a hemoglobin level of 6.9 g/dL. After receiving intravenous antibiotics and beginning transfusion of one unit of packed red blood cells, the patient went from being weak on examination to becoming lethargic, hypoxic, hypercapnic, hypertensive, and hypothermic due to flash pulmonary edema in the setting of a hypertensive emergency. Within only a few hours of being in the emergency department, the patient required transfer to the medical ICU; a prolonged hospital course ensued, eventually leading to a stable discharge, nearly three months later.

## Introduction

Systemic sclerosis (SSc), commonly referred to as scleroderma, is a chronic autoimmune disease characterized by fibrosis, vascular dysfunction, and immune dysregulation affecting the skin and multiple internal organ systems [1]. Scleroderma can be divided into two primary forms: localized scleroderma and SSc. SSc can be further divided into limited SSc (formerly known as CREST syndrome) and diffuse SSc [2]. Localized scleroderma primarily affects the skin and subcutaneous tissue, whereas SSc is associated with systemic manifestations and internal organ involvement. Although uncommon, with an estimated prevalence of approximately 20 cases per million people in the United States, SSc carries significant morbidity and mortality due to pulmonary, gastrointestinal, renal, and cardiac involvement [1].

Diffuse SSc is particularly associated with progressive internal organ involvement, including interstitial lung disease (ILD), pulmonary hypertension, gastrointestinal dysmotility, and scleroderma renal crisis [3,4]. Cumulative survival from the time of diagnosis of SSc has been estimated at 74.9% at 5 years and 62.5% at 10 years [5]. This corresponds to a 5-year mortality rate of roughly 25% and a 10-year mortality rate of 37-38%. Pulmonary involvement represents the main cause of death. Because many healthcare providers outside of rheumatology and dermatology may encounter this disease infrequently, delayed recognition and treatment may contribute to adverse outcomes.

We present a case of diffuse SSc complicated by ILD, aspiration pneumonia, hypertensive emergency, and flash pulmonary edema requiring intensive care unit (ICU)-level care.

## Case presentation

A 53-year-old female patient with a history of SSc diagnosed in 2023 and complicated by ILD requiring 2 L of home oxygen, gastric antral vascular ectasia, and prior scleroderma renal crisis presented to the emergency department with progressively worsening dysphagia. Although symptoms had been present for approximately one year, the patient reported significant worsening over the preceding two weeks with markedly decreased oral intake.

Three days prior to presentation, the patient underwent upper endoscopy, which demonstrated biopsy proven esophageal candidiasis, and she had recently started fluconazole therapy. Due to worsening dysphagia and inability to tolerate oral intake, she was advised by her gastroenterologist to seek emergency care.

Her prior medical history was notable for significant difficulty obtaining consistent outpatient treatment due to medication intolerance and insurance-related barriers. She had previously been diagnosed with SSc after presenting with joint pain, finger swelling, and stiffness. Earlier CT imaging demonstrated interstitial fibrosis, and she was initially treated with mycophenolate mofetil, which was discontinued because of urticaria. Tocilizumab was later considered but delayed due to insurance limitations and ultimately discontinued after a lapse in insurance coverage. Methotrexate therapy was attempted but poorly tolerated because of worsening joint pain and cough.

Pulmonology evaluation prior to admission had included consideration of antifibrotic therapy and lung transplantation evaluation. At the time of admission, the patient was not receiving active immunosuppressive therapy. Intravenous immunoglobulin (IVIG) therapy had been planned in the outpatient setting but had not yet been initiated prior to hospitalization.

On presentation, the patient was hypertensive with systolic blood pressures greater than 170 mmHg. Laboratory studies were notable for symptomatic anemia with a hemoglobin level of 6.9 g/dL (reference range: 12.5-16.0 g/dL) and elevated high-sensitivity troponin I levels of 1,888 ng/L (reference range: 0-53 ng/L). Chest radiography demonstrated bilateral pulmonary opacities concerning for multifocal and aspiration pneumonia (Figure 1). Computed tomography (CT) imaging of the chest without contrast demonstrated extensive interstitial fibrosis and bronchiectatic changes (Figure 2). 

**Figure 1:**
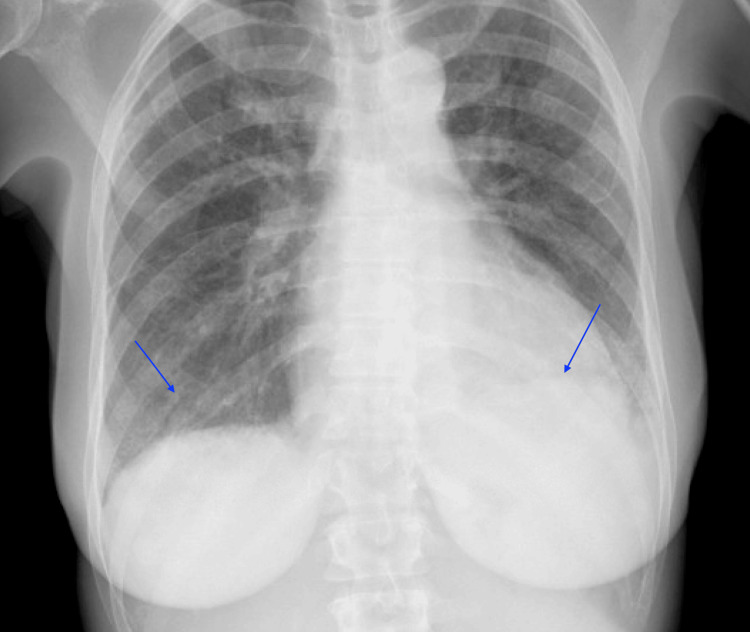
Initial chest radiograph demonstrating bilateral pulmonary opacities concerning for multifocal and aspiration pneumonia. Opacities on chest radiograph are visualized by areas of haziness, which appear lighter and often indicate increased lung density. This may be seen in pleural effusions, masses, or infections such as pneumonia. In this case, the increased haziness is structured more towards the lower lobes, with the left being greater than the right.

**Figure 2:**
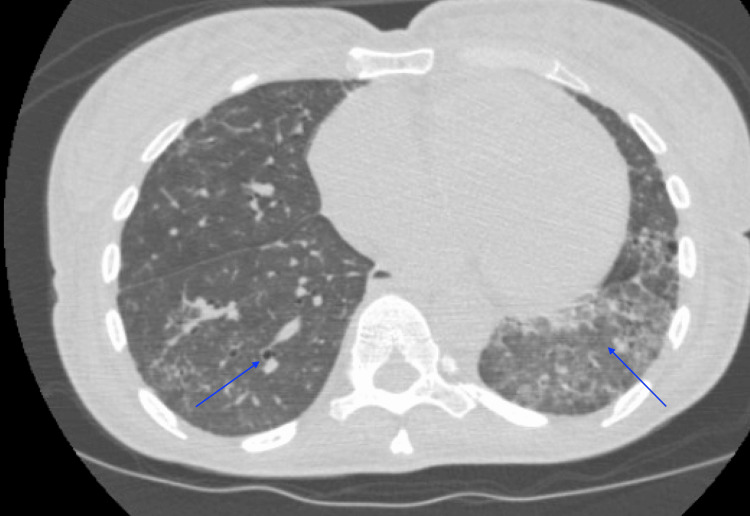
Computed tomography of the chest without contrast demonstrating extensive interstitial fibrosis and bronchiectatic changes. Interstitial fibrosis is abnormal scarring and thickening of lung tissue, which sometimes presents with a honeycomb-like appearance. This is one of the major complications seen in patients with systemic scleroderma. Interstitial fibrosis can also lead to traction bronchiectasis. Traction bronchiectasis refers to the stretching and widening of airways caused by surrounding fibrosis.

The patient was initially started on cefepime 2 grams intravenously and vancomycin 1 gram intravenously and transfused one unit of packed red blood cells. Shortly after the initiation of transfusion, she developed worsening dyspnea and acute respiratory distress. Arterial blood gas analysis revealed severe acidosis with a pH of 7.18 (reference range: 7.35-7.45). Repeat chest radiography demonstrated worsening bilateral pulmonary opacities and vascular congestion consistent with flash pulmonary edema (Figure 3).

**Figure 3:**
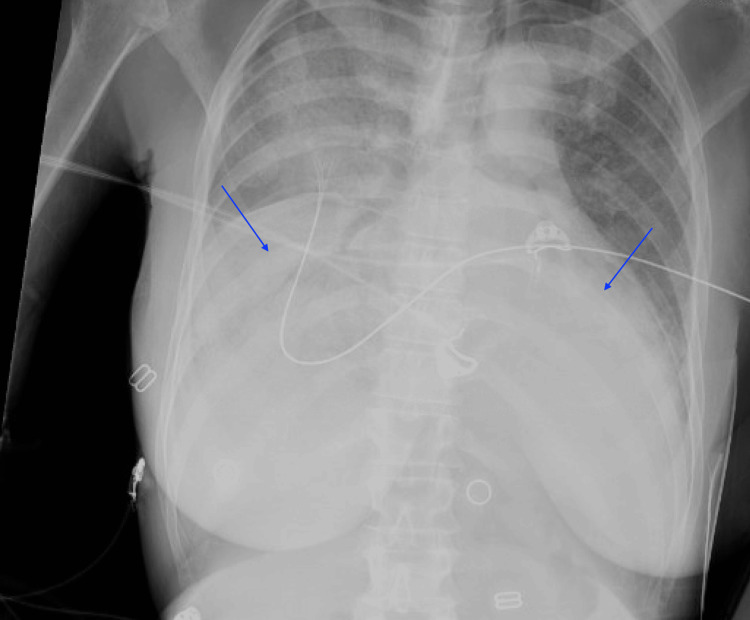
Repeat chest radiograph demonstrating worsening bilateral pulmonary opacities and pulmonary vascular congestion consistent with flash pulmonary edema. Worsening airspace opacities are now seen. The right lung has more significant involvement compared to the left lung. This can be seen in the setting of pleural fluid or an infection such as pneumonia. This chest radiograph was taken only a few hours after the initial one. This leads us to believe that these worsening opacities are due to acute fluid build-up, which led to acute hypoxic respiratory failure. This could be a complication of scleroderma renal crisis.

The patient rapidly became hypoxic and hypercapnic and required escalation of care with bilevel positive airway pressure (BiPAP), intravenous nicardipine infusion for hypertensive emergency management, and transfer to the medical ICU. While in the ICU, the patient was transitioned to intravenous metoprolol tartrate and intravenous enalaprilat.

The patient experienced a prolonged hospitalization lasting nearly three months due to recurrent aspiration with an associated pulseless electrical activity (PEA) arrest, difficulty with handling high-residual tube feeds, tracheostomy and ventilator dependence, recurrent infections, and ongoing palliative care discussions, with an eventual stable discharge to a long-term care facility. Review of the medical record demonstrated readmission to another hospital less than one month later for acute hypoxic respiratory failure secondary to pneumonia. During her hospitalization, IVIG therapy was initiated with plans for consideration of outpatient mycophenolic acid therapy.

## Discussion

SSc is a chronic autoimmune multisystem disorder characterized by inflammatory, vascular, and fibrotic changes involving the skin and internal organs [1]. Although the exact etiology remains unclear, current evidence suggests a multifactorial disease process involving endothelial injury, immune dysregulation, cytokine release, autoantibody formation, and fibroblast activation leading to excessive connective tissue deposition and fibrosis [4].

SSc is generally classified into limited cutaneous SSc and diffuse cutaneous SSc. Limited disease commonly affects the fingers, hands, forearms, and face, whereas diffuse disease is associated with extensive internal organ involvement, including pulmonary fibrosis, gastrointestinal dysmotility, cardiac involvement, and renal crisis [6].

Pulmonary complications remain among the leading causes of mortality in SSc, particularly ILD and pulmonary arterial hypertension [7]. Gastrointestinal manifestations, including dysphagia and esophageal dysmotility, are also common and can contribute to aspiration events and malnutrition. This patient demonstrated severe multisystem disease with pulmonary fibrosis, recurrent aspiration, prior renal crisis, and significant gastrointestinal involvement.

This case also highlights the challenges associated with treatment access and medication intolerance in SSc management. It is also important to note that the 2023 American College of Rheumatology (ACR) guidelines for the treatment of ILD in people with systemic autoimmune rheumatic disease (SARD) differ based on whether the patient has SARD with ILD or whether the patient specifically has SSc with ILD [7]. For patients with SARD-ILD other than SSc-ILD, glucocorticoids are conditionally recommended as a first-line ILD treatment. However, for people with SSc-ILD, the ACR strongly recommends against glucocorticoids as a first line ILD treatment. This is because in patients with SSc-ILD, glucocorticoids can increase the risk of scleroderma renal crisis. For patients with SSc-ILD and mixed connective tissue ILD, the ACR conditionally recommends tocilizumab as a first-line ILD treatment option. For patients with SSc-ILD, the ACR conditionally recommends nintedanib as a first-line ILD treatment option. These guidelines highlight the complexity of medical decision regarding treatment options for SSc-ILD. Patients with severe diffuse disease or progressive pulmonary involvement should ideally be managed at specialized centers with expertise in SSc.

This patient’s hospital course illustrates how rapidly SSc-related complications, such as ILD, can progress and how multiple overlapping pathologies, including aspiration pneumonia, severe anemia, hypertensive emergency, pulmonary edema, and chronic ILD, can lead to critical illness requiring ICU-level care.

## Conclusions

SSc is a rare but potentially devastating autoimmune disease with significant multisystem involvement and high morbidity. This case demonstrates the complexity of advanced SSc complicated by ILD, gastrointestinal dysmotility, hypertensive emergency, and acute respiratory failure requiring ICU-level care. Early recognition of organ involvement, multidisciplinary management, timely access to immunosuppressive therapy, and close outpatient follow-up are essential to improving outcomes in patients with diffuse SSc.
